# The First Report of Target-Site Resistance to Glyphosate in Sweet Summer Grass (*Moorochloa eruciformis*)

**DOI:** 10.3390/plants10091885

**Published:** 2021-09-11

**Authors:** Romesh Salgotra, Bhagirath Singh Chauhan

**Affiliations:** 1 Queensland Alliance for Agriculture and Food Innovation (QAAFI), The University of Queensland, Gatton, QLD 4343, Australia; rks_2959@rediffmail.com; 2 School of Biotechnology, Sher-e-Kashmir University of Agricultural Sciences & Technology of Jammu, Chatha 180009, India; 3 Department of Agronomy, Chaudhary Charan Singh Haryana Agricultural University (CCSHAU), Hisar 125004, India

**Keywords:** sweet summer grass, molecular mechanism, target-site resistance, glyphosate, double point mutation

## Abstract

Sweet summer grass is a problematic weed in the central Queensland region of Australia. This study found glyphosate resistance in two biotypes (R1 and R2) of sweet summer grass. The level of resistance in these biotypes was greater than 8-fold. The glyphosate dose required to reduce dry matter by 50% (GR50) for the resistant populations varied from 1993 to 2100 g ha^−1^. A novel glyphosate resistance double point mutation in the 5-enolpyruvylshikimate-3-phosphate synthase (EPSPS) gene was identified for the first time in sweet summer grass. Multiple mutations, including multiple amino acid changes at the glyphosate target site, as well as mutations involving two nucleotide changes at a single amino acid codon, were observed. Both resistant biotypes exhibited a nucleotide change of CAA to ACA in codon 106, which predicts an amino acid change of proline to a threonine (Pro-106-Thr). In addition, the R1 biotype also possessed a mutation at codon 100, where a nucleotide substitution of T for G occurred (GCT to TCT), resulting in a substitution of serine for alanine (Ala-100-Ser). Understanding the molecular mechanism of glyphosate resistance will help to design effective management strategies to control invasive weeds.

## 1. Introduction

**Figure 1:**
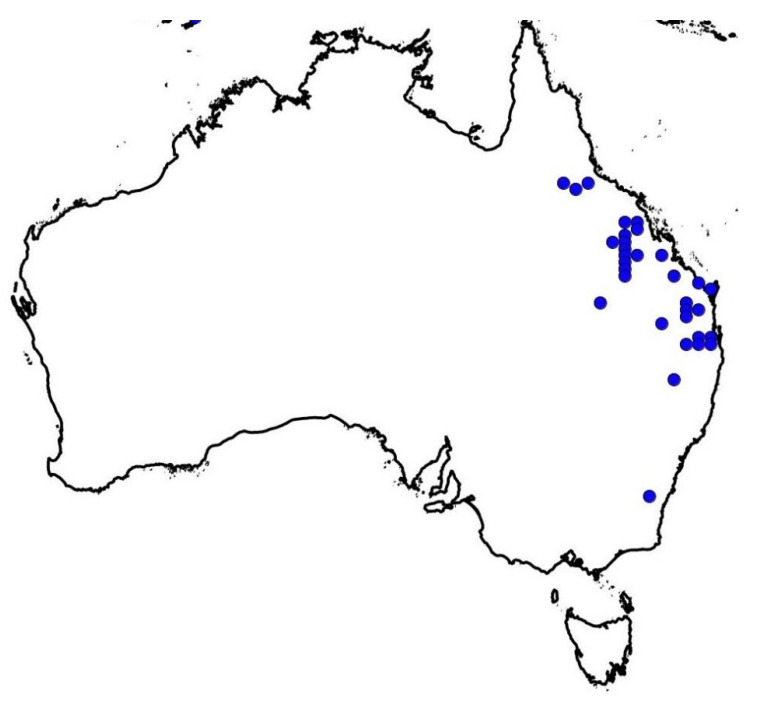
Sweet summer grass-infested areas of Australia (https://avh.ala.org.au/occurrences/search?taxa=Moorochloa+eruciformis#tab_mapView, accessed on 11 September 2021).

Sweet summer grass (SSG) (*Moorochloa eruciformis* (Sm.) Veldkamp) (synonym = *Brachiaria eruciformis* (Sm.) Griseb.) is a noxious weed of field crops and fallow areas in Australia (Figure 1), particularly in the sorghum and cotton-growing areas of Queensland and New South Wales [1]. The problematic weed is also found in the tropic and sub-tropic zones of Africa, the Americas, Asia, and some parts of Europe [2,3,4]. It is a summer dominant weed; however, this weed is often present in the late phases of winter crops. SSG is hardy and poses a significant problem in cotton crops cultivated under dryland conditions compared to irrigated conditions [1]. After harvesting the summer crop, the remnant of weed impedes the emergence of winter crops. It forms thick grass mats in the cropped areas, which interfere and cause a significant crop yield loss [5,6,7].

SSG is an annual weed that belongs to the family Poaceae. It is a fine and delicate weed compared to other prevalent summer weeds. This weed gives a sprawling stoloniferous look where lower joints of roots touch the ground. The plants grow up to the height of 60 cm. Leaves are 10–15 cm long, simple, and arranged alternatively on the plant. Short hairs are present on the clumps of leaves and are cylindrical. The plant has dark green blades and reddish-purple tinges, particularly around the leaf margin and sheath. The panicle of the plant is erect on the main axis with 2–12 lateral branches. Although SSG is a short-lived summer weed, it is easily dispersed by seeds to different places. The seed production potential of this weed is more than 100,000 seeds per plant. The high rate of seed production, short generation time, and the high competitiveness are the main factors leading to the evolution of this species as a major weed [1].

To control SSG in fallows and glyphosate-tolerant crops, glyphosate is the most widely used non-selective post-emergence herbicide. Glyphosate targets 5-enolpyruvylshikimate-3-phosphate synthase (EPSPS) [1], a key enzyme in the aromatic amino acid biosynthesis pathway in plants, fungi, and bacteria [8]. Widespread application of herbicides has spurred the rapid and repeated evolution of herbicide resistance in agricultural weeds, and at least 43 weed species have evolved glyphosate resistance [8]. It provides inexpensive, flexible, simple, and effective control of a broad spectrum of weeds. Crops can be sown shortly after the application of glyphosate as this herbicide has no residual soil activity [9]. In Australia, the intensive use of glyphosate to control this weed has led to the appearance of glyphosate-resistant SSG at a number of sites. Moreover, the out-crossing of glyphosate-resistant biotypes with susceptible biotypes may lead to the evolution of more resistant biotypes. The adjacent areas to the cropped fields, such as irrigation channels, roadways, fence lines, tail drains, and areas next to stock routes, can be a significant entry source for resistant weed seeds [10].

The knowledge of specific DNA changes conferring non-target-site resistance is still in its early stages, while the detection of the first DNA change conferring evolved target-site resistance was determined over four decades ago [11,12]. Since then, several targeted-site mutations conferring herbicide resistance have been identified in several weed species [13]. Glyphosate resistance mechanisms in weeds are either by target-site mutation alterations or non-target-site mechanisms. In target-site mutation alterations, amino acids substitutions occur that subsequently affect the glyphosate interaction at the EPSPS enzyme [7]. However, in target-site alterations, increased EPSPS gene amplification may also occur, which produces sufficient protein to operate the shikimate pathway [8,14]. In the non-target-site mechanism, there is reduced uptake of glyphosate [15,16], reduced glyphosate translocation [17,18], and/or vacuole sequestration where glyphosate is sequestered in the cell vacuole of resistant plants [19]. The first case of glyphosate-resistant SSG in Australia was reported in 2014; however, the mechanism of resistance to glyphosate has not been elucidated in this weed species [8]. Australian growers are reporting difficulty in controlling this weed despite several glyphosate sprays. Therefore, there is a need to understand the mechanism of glyphosate resistance development so that management strategies can be developed to control this invasive weed. By understanding the mechanism of herbicide resistance, strategies can be developed both to slow the evolution of resistance and to control existing resistant biotypes [20]. This study documents the first known case of field-evolved target-site resistance to glyphosate in Australian biotypes of SSG. Hence, the objective of this study was to identify the molecular mechanism of glyphosate resistance in SSG.

## 2. Results

### 2.1. Response to Glyphosate Dose

**Figure 2:**
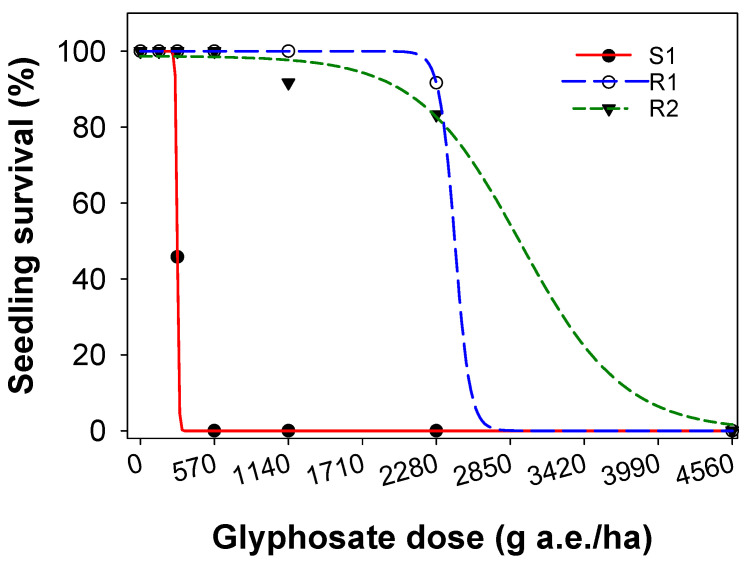
Effect of glyphosate dose on survival (%) of a glyphosate-susceptible (S1) and two glyphosate-resistant (R1 and R2) biotypes of sweet summer grass. A three-parameter sigmoid model fitted to the survival data.

**Figure 3:**
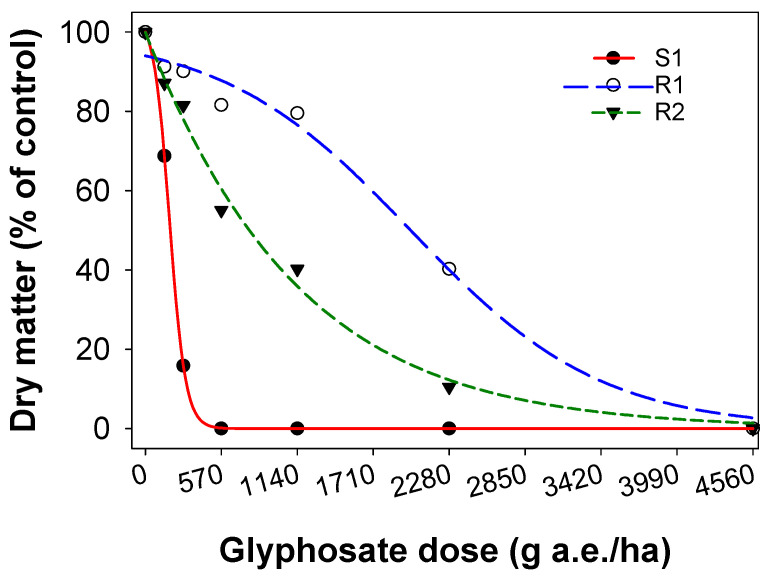
Effect of glyphosate dose on dry matter (percent of non-treated control) of a glyphosate-susceptible (S1) and two glyphosate-resistant (R1 and R2) biotypes of sweet summer grass. A three-parameter sigmoid model fitted to the dry matter data.

The dose–response study confirmed resistance in two SSG biotypes (R1 and R2) to glyphosate herbicide. The glyphosate dose required to kill 50% of the plants (LD_50_) was 284 g a.e. ha^−1^ (g ha^−1^, hereafter) for the S1 biotypes (Table 1; Figure 2). The LD_50_ for the two resistant biotypes ranged from 2421 to 2921 g ha^−1^, which was 8.5 to 10.2-fold greater than the S1 biotype. The glyphosate dose required to reduce dry matter by 50% (GR_50_) was 182 g ha^−1^ for the susceptible biotype (Table 1; Figure 3). The GR_50_ for the resistant biotypes ranged from 1993 to 2100 g ha^−1^, which was 11.0 to 11.5 times greater than the susceptible biotype.

### 2.2. EPSPS Gene Sequencing

Target-site mutations for glyphosate resistance were identified in the resistant biotypes of SSG. The nucleotide sequences of the susceptible biotype differed from that of the resistant biotypes (Table 2). Comparisons of the sequences of each biotype revealed that both resistant biotypes (R1 and R2) possessed a mutation at codon 106, where a nucleotide substitution of A for C occurred, resulting in a target-site EPSPS mutation by substitution of a threonine amino acid for proline (Pro-106-Thr).

Both resistant biotypes (R1 and R2) exhibited a nucleotide change of CAA to ACA in codon 106, which predicts an amino acid change of proline to a threonine (Pro-106-Thr). In addition, biotype R1 also possessed a mutation at codon 100, where a nucleotide substitution of T for G occurred (GCT to TCT), resulting in a replacement of alanine by serine. In the sequencing, both resistant biotypes of SSG possessed silent mutation at p105. The silent mutation resulted in the substitution of CGG to CGA. There are reports that the additional point mutations also occur in addition to Pro-106. In addition, a glyphosate resistance mutation at point 100 (p100) (alanine to serine at p100) was also observed in the R1 biotype. In the R2 biotype, only the one mutation at p106 was observed. In the study, a double point mutation (threonine amino acid for proline at p106 and alanine to serine at p100) was found in SSG. It has been observed that the reported mutations conferring glyphosate resistance in weeds change the hydrophobic Pro106 amino acid of EPSPS to the amino acid threonine. However, sometimes the Pro106 is not directly involved in molecular interactions with glyphosate, but the available space within the active site is reduced by changing the Pro106 to a different amino acid, causing a structural change in the active site. Technically, the Pro106 change causes a steric hindrance of the glyphosate molecule to the binding site.

## 3. Discussion

High selection pressure on herbicide-resistant weeds occurs due to the continuous application of a single herbicide to control weeds. The rapid diagnosis of herbicide-resistance mutations in weed biotypes helps in the formulation of a strategy to control invasive weeds. The causal variants for herbicide resistance have largely been considered to be the result of a single nucleotide change from the wild-type genome. Most of the herbicide resistance mechanisms are characterized through a single modification of nucleotide change except for the double substitution nucleotide changes in EPSPS gene in goosegrass (*Eleusine* *indica*) [22]. The primary goal motivating the need to characterize herbicide resistance mechanisms is to frame a strategy for the management of herbicide-resistant weeds, which is mostly dependent on molecular mechanisms and the distribution of the resistance mechanism. In this study, we present the molecular mechanism to detect herbicide resistance in SSG biotypes.

The dose–response study confirmed the resistance in two SSG biotypes (R1 and R2) to glyphosate. Very high levels of glyphosate resistance have been reported in weeds such as jungle rice (*Echinochloa colona*), rigid ryegrass (*Lolium rigidum*), horseweed (marestail; *Conyza canadensis*), Italian ryegrass (*Lolium multiflorum*), *Amaranthus* species, goosegrass, hairy fleabane (*Conyza bonariensis*), and Johnsongrass (*Sorghum halepense*), etc. [8,23,24]. The exposure of SSG biotypes to glyphosate has provided the selection pressure required for the evolution of resistance.

To minimize confusion about the convergent evolution of this point mutation, we adhered to the standard amino acid numbering system by Baerson et al. [21]. In the mature *EPSPS* enzyme of plants, amino acids are one of the two known conserved regions of EPSPS located between positions 90 and 110, and between 175 and 200 [25]. In several plant species, the substitutions of amino acid at positions 101, 102, 106, 144, and 192 can be introduced into the conserved regions of wild-type EPSPS enzymes to yield glyphosate-tolerant EPSPS enzymes [25,26]. Similarly, in *Escherichia coli* and *Salmonella typhimurium*, a highly conserved region of EPSPS was labeled from amino acids 86 to 131 [27]. More than 40 species have evolved resistance to glyphosate, and at least 13 species have a target-site mutation at position 106 of EPSPS [28,29,30,31]. Recently, a target-site mutation was reported in two biotypes (SGM2 and SGW2) of feathertop Rhodes grass (*Chloris virgata*), in which threonine substitution for proline occurred [32].

In our study, a double point mutation (threonine amino acid for proline at p106 and alanine to serine at p100) was found. We suggest that the recent discovery of the p106 mutation conferring glyphosate resistance in SSG could be evolved with more resistance due to the use of high doses of glyphosate to control this weed. Both resistant biotypes (R1 and R2) showed similar mutations at p106 by substitution of a threonine amino acid for proline (Pro-106-Thr), whereas two mutations occurred in biotype R1. This could be due to glyphosate herbicide selection pressure, gene flow, and geographical isolations. A similar response has also been observed in other weed species [33,34,35].

The novel glyphosate resistance double point mutation in the EPSPS gene has been identified for the first time in SSG in Australia. Similarly, individuals of gooseweed from Malaysia, Australia, and China, and beggarticks (*Bidens Pilosa*) from Mexico have also reported the double point mutations for herbicide resistance [22,36]. The double point mutation in the nucleotide sequences is associated with increased glyphosate resistance in weeds. The first report of a double EPSPS mutation in glyphosate-resistant weed biotype has demonstrated the evolutionary process by which mutations can accumulate to confer increasingly efficient resistance when selection pressure is persistent [30]. Recently, a triple point mutation (threonine to isoleucine at p102, alanine to valine at p103, and proline to serine at p106) was found to be the glyphosate resistance mechanism within individuals of smooth amaranth (*Amaranthus hybridus*) from Argentina [37]. 

To our knowledge, these findings represent the first documentation of target-site mediated glyphosate resistance in SSG. Single point mutations are sufficient to affect the binding of glyphosate to the EPSPS gene, resulting in herbicide resistance. Generally, in herbicide resistance, there are four common amino acid substitutions for these mutations, such as alanine, proline, tryptophan, and serine [31]. Once herbicide resistance has been developed in weeds, it is widely spread to the susceptible plants by outcrossing. Sometimes, out-crossing of the glyphosate-resistant weeds with susceptible genotypes leads to an increased number of herbicide-resistant biotypes. The resulting new biotypes are difficult to control. The glyphosate-resistant weeds spread speedily in cropping fields through roads, irrigation channels, tail drains, etc. The areas next to stock routes can be a significant entry source for resistant weed seeds [9]. Besides this, the other major factors contributing to the rapid spread of resistance are: (i) pollen mediated gene flow allowing for resistance in plants that have never been exposed to the herbicide, (ii) very strong herbicide selection pressure, (iii) no fitness penalty associated with the resistance gene, and (iv) a dominant trait resulting in highly resistant heterozygous individuals [31].

The evolution to high-level glyphosate resistance in SSG could be due to the double point mutation (threonine amino acid for proline at p106 and alanine to serine at p106). The knowledge of the molecular basis of the mechanism of herbicide resistance helps to develop strategies both to control resistance in existing biotypes and to slow down the evolution of herbicide resistance in weeds. However, to control glyphosate-resistant SSG, some effective measures, such as herbicide mixtures or rotations with different weed control mechanisms, need to be undertaken. Besides this, herbicide mixtures and other management programs, such as removal of weed seeds, crop rotation, and the cleaning of machinery, can help to control the spread and development of glyphosate-resistant weeds.

## 4. Materials and Methods

### 4.1. Seed Collection

Mature seeds of SSG were collected in June 2020 from fields in Central Queensland, Australia. The S1 biotype was collected from a sorghum crop (−23.2016, 148.2863; Capella) cultivated under organic farming. The R1 and R2 biotypes were collected from fallow fields (R1, −23.2387, 148.0876, Gordonstone; and R2, −22.8566, 147.9074, Clermont). Seeds were collected by shaking mature plants over trays, which were then cleaned and stored in ambient conditions until the start of the experiment. The study was conducted at the weed science research facility of the Queensland Alliance for Agriculture and Food Innovation (QAAFI), The University of Queensland, Gatton, Australia.

### 4.2. Response to Glyphosate Dose

Seeds (12) of the three biotypes (R1, R2, and S1) were planted in pots (12 cm diameter) containing a commercial potting mix media. Immediately after emergence, plants were thinned to keep only 4 plants/pot. Plants were grown outdoors and watered as required. Glyphosate was applied to plants at the 4–5 leaf stage. Seven different doses of glyphosate (0, 143, 285, 570, 1140, 2280, and 4560 g a.e. ha^−1^) were applied with the help of a research track sprayer (manufactured by Woodlands Road Engineering, Gatton, QLD 4343, Australia), which delivered 108 L ha^−1^ spray volume through flat fan nozzles (TeeJet XR 110015). Plant survival data was recorded at 28 days after treatment with the survival criterion of at least one new leaf forming after herbicide application. Plants that survived were harvested at the surface level and dried in an oven at 70 °C for 72 h and weighed. The experiment was conducted using a randomized complete block design with three replications. The study was repeated once. The difference between the experimental runs was non-significant, and no interaction was observed between the experimental run and treatments [38]. Therefore, the data was pooled over the experimental run.

The dry matter of all populations was expressed as a percentage of the non-treated control treatment for that population. LD50 (glyphosate dose required to kill 50% of the plants) and GR50 (glyphosate dose required to reduce shoot biomass by 50%) were calculated by fitting a three-parameter sigmoid model to the survival and dry matter data, respectively (SigmaPlot 14.0). The fitted model was

*Y* = *a*/(1 + e [−(*x* − *G_50_*)/*b*])

where *Y* is the seedling survival or seedling dry biomass at glyphosate dose *x*, *a* is the maximum seedling survival or seedling dry biomass, *G_50_* is the glyphosate dose (g a.e. ha^−1^) required for 50% reduction in plant survival or shoot biomass, and *b* is the slope of the curve. Resistance index was calculated as the ratio between the LD50 or GR50 of each resistant population and the LD50 or GR50 of the susceptible population.

### 4.3. DNA Extraction

Plants of the S1 biotype were chosen from the control (non-treated) treatment, whereas survived plants (glyphosate 2280 g ha^−1^) of the resistant biotypes (R1 and R2) were chosen for extraction of DNA [39]. Total genomic DNA was extracted from 100 mg of frozen leaf tissue using DNeasy Plant Mini Kits (Qiagen, USA) according to the manufacturer’s protocol [40]. The quality of the isolated DNA of all the samples was checked on a 1% agarose gel. The concentration and relative purity of the isolated DNA was checked using Nanodrop ND-1000 (Agricultural Genomics Laboratory). DNA samples were diluted with distilled water and adjusted to 30 ng μL^−1^. The DNA samples were stored at −20 °C for further studies [39].

### 4.4. EPSPS Gene Sequencing

After the extraction of genomic DNA from leaf tissue with a DNeasy Plant Mini Kit (QIAGEN) following manufacturer instructions. A polymerase chain reaction was performed to amplify the conserved region of the EPSPS gene. Primer sequences were the same as those used for EPSPS sequencing in Ngo et al. [31] (Forward—AACAGTGAGGAYGTYCACTACATGCT; Reverse—CGAACAGGAGGGCAMTCAGTGCCAAG). Each PCR was set up with a total volume of 20 µL, containing 4 µL 5x MyTaq reaction buffer (Bioline, Eveleigh, NSW, Australia), 30 ng template DNA, 0.3 µM of each primer, 0.2 µL MyTaq HS DNA polymerase (Bioline, Eveleigh, NSW, Australia), and 13.6 µL H_2_O. All reactions were performed in a T100 Thermal Cycler (Bio-Rad, Gladesville, NSW, Australia) with cycle conditions as follows: 3 min denaturing at 95 °C; 35 cycles of 30 s denaturation at 95 °C, 30 s annealing at 57 °C, 45 s elongation at 72 °C, and a final extension for 7 min at 72 °C. Samples were electrophoresed in 1 × TAE buffer (pH to 8 with glacial acetic acid) at 120 V and photographed under UV light (λ = 302 nm). DNA fragment sizes were determined by comparing with a 100 bp DNA ladder (Bioline) and the ethidium bromide-stained gels were documented using GelRed Documentation System (Biotium, Fremont, CA, USA). DNA sequencing of the PCR products was carried out with the same primers used for amplification at the Australian Genome Research Facility Ltd. (AGRF), Australia. Sequences were checked for quality and an alignment was employed to compare sequencing data of a susceptible biotype, two resistant biotypes, and the known *EPSPS* susceptible sequences of sweet summer weed using Molecular Evolutionary Genetics Analysis (MEGA) software [41].

## 5. Conclusions

The detection of a new mechanism of herbicide resistance in weeds is important to increase the efficacy of herbicides and to plan effective strategies to control weeds. Glyphosate resistance is a growing problem in Australia, particularly in Queensland and New South Wales states, with concerns several more weed species may be on the cusp of becoming resistant. Recently, incidences of glyphosate failure on SSG have been reported in Australia, specifically in farmers’ fields. Two glyphosate-resistant biotypes (R1 and R2) of SSG collected from Queensland were compared with one susceptible (S1) biotype to confirm glyphosate resistance and elucidate possible mechanisms of resistance. In SSG, resistance to glyphosate was primarily due to target-site EPSPS mutation (Pro-106-Thr). In addition, a double point mutation (Pro-106-Thr and Ala-100-Ser) has occurred in the R1 biotype. In conclusion, this study has identified high levels of glyphosate resistance in two SSG biotypes. This research is the first to report the evolution of an EPSPS double mutation conferring very high-level glyphosate resistance in crop fields.

## Figures and Tables

**Table 1:** Estimated glyphosate dose required to kill 50% of the plants (LD_50_), glyphosate dose required to reduce dry matter by 50% (GR_50_), and resistance indices (RI).

Population	LD_50_	RI	GR_50_	RI
S1	284		182	
R1	2421	8.5	1993	11.0
R2	2921	10.2	2100	11.5

RI were calculated as the ratio between the LD50 or GR50 of each resistant population and the LD50 or GR50 of the susceptible control.

**Table 2:** Aligned nucleotide sequences of the conserved region of EPSPS for three biotypes of *Brachiaria eruciformis*. Codons are numbered according to Baerson et al. [21]. ^1^ Silent mutation.

Amino Acid Number:	96	97	98	99	100	101	102	103	104	105	106	107	108
Amino acid:	Phe	Leu	Gly	Asn	Ala	Gly	Thr	Ala	Met	Arg	Pro	Leu	Thr
Sequence:	TTC	TTG	GGG	AAT	GCT	GGA	ACT	GCA	ATG	CGG	CCA	TTG	ACA
S1	-	-	-	-	-	-	-	-	-	-	-	-	-
R1	-	-	-	-	TCT	-	-	-	-	CGA ^1^	ACA	-	-
R2	-	-	-	-	-	-	-	-	-	CGA ^1^	ACA	-	-

## Data Availability

All data is available within the manuscript.
